# Pharmacokinetics of Sublingually Delivered Fentanyl in Head and Neck Cancer Patients Treated with Curatively Aimed Chemo or Bioradiotherapy

**DOI:** 10.3390/cancers10110445

**Published:** 2018-11-15

**Authors:** Evelien J. M. Kuip, Wendy H. Oldenmenger, Esther Oomen-de Hoop, Gerda M. Verduijn, Martine F. Thijs-Visser, Peter de Bruijn, Esther van Meerten, Stijn L. W. Koolen, Ron H. J. Mathijssen, Carin C. D. van der Rijt

**Affiliations:** 1 Department of Medical Oncology, Erasmus MC Cancer Institute, Dr. Molewaterplein 40, 3015 GD Rotterdam, The Netherlands; w.h.oldenmenger@erasmusmc.nl (W.H.O.); e.oomen-dehoop@erasmusmc.nl (E.O.-d.H.); m.f.thijs@ikazia.nl (M.F.T.-V.); p.debruijn@erasmusmc.nl (P.d.B.); e.vanmeerten@erasmusmc.nl (E.v.M.); s.koolen@erasmusmc.nl (S.L.W.K.); a.mathijssen@erasmusmc.nl (R.H.J.M.); c.vanderrijt@erasmusmc.nl (C.C.D.v.d.R.); 2 Department of Medical Oncology, Radboud University Medical Center, Geert Grooteplein Zuid 8, 6500 HB Nijmegen, The Netherlands; 3 Department of Anesthesiology, Pain and Palliative Medicine, Radboud University Medical Center, Geert Grooteplein Zuid 10, 6500 HB Nijmegen, The Netherlands; 4 Department of Radiation Oncology, Erasmus MC Cancer Institute, Dr. Molewaterplein 40, 3015 GD Rotterdam, The Netherlands; g.verduijn@erasmusmc.nl; 5 Department of Hospital Pharmacy, Erasmus MC, University Medical Center, Dr. Molewaterplein 40, 3015 GD Rotterdam, The Netherlands; 6 Netherlands Comprehensive Cancer Organisation, Godebaldkwartier 419, 3511 DT Utrecht, The Netherlands

**Keywords:** fentanyl, mucositis, pain, pharmacokinetics, radiotherapy, xerostomia

## Abstract

Over 90% of patients treated for head and neck cancer with curatively aimed chemo or bioradiotherapy will develop painful mucositis and xerostomia. Sublingually delivered fentanyl (SDL) is a rapid acting opioid to treat breakthrough pain. It is unclear how SDL is absorbed by the mucosa of these patients. Therefore, the aim of this study was to investigate the effects of mucositis and xerostomia on the absorption of SDL. Thirteen patients who received chemo or bioradiotherapy (RT), were given a single dose of fentanyl: Before start of RT, 3 and 6 weeks after start of RT, and 6 weeks after finishing RT. Pharmacokinetic samples were taken. The primary endpoint was the relative difference (RD) between systemic exposure to fentanyl (area under the curve; AUC) at baseline (AUC_baseline_) and fentanyl AUC in the presence of mucositis grade ≥2. The secondary endpoint was the RD between AUC_baseline_ and fentanyl AUC in the presence of xerostomia, which were analyzed by means of a paired *t*-test on log-transformed data. Mucositis resulted in a 12.7% higher AUC (*n* = 13; 95% CI: −10.7% to +42.2%, *p* = 0.29) compared to baseline levels and xerostomia resulted in a 22.4% lower AUC (*n* = 8; 95% CI: −51.9% to +25.3%, *p* = 0.25) compared to baseline levels. Mucositis grade ≥2 or xerostomia caused by chemo or bioradiotherapy does not significantly alter the systemic exposure to SDL. Patients with pain during and after chemo or bioradiotherapy may be safely treated with SDL.

## 1. Introduction

For patients with head and neck cancer, combined treatment strategies consisting of radiotherapy and cisplatin (chemoradiotherapy) or cetuximab (bioradiotherapy), respectively, have been reported with improved local tumor control and survival rate in comparison to radiotherapy alone [1,2,3]. The consequence of this combined treatment, however, is a higher incidence of severe and dose-limiting side effects during and after therapy. This is especially the case for mucositis and xerostomia [1,2,3,4].

Mucositis is an inflammatory process of the mucosa characterized by erythema, inflammation, and/or ulceration of the mucosa due to tissue damage [4,5]. Reported overall incidence rates of mucositis after chemoradiotherapy or bioradiotherapy are 97% and 93%, and for common terminology criteria (CTC) grade ≥3 mucositis 34% and 56%, respectively [6]. Mucositis may be associated with severe pain, weight loss, need for a feeding tube, hospitalization, and, as a result, increased medical costs [7]. Especially, cumulative doses of >39 Gy were associated with severe mucositis [8].

During combined therapy, the severity of mucositis gradually increases, and most patients require analgesics—mostly opioids—from the third week of treatment until 2 to 6 weeks after the last radiotherapy dose [4,9]. Due to mucositis and associated swallowing problems, the use of oral medication can be difficult and painful [10,11]. Therefore, transdermal opioids are preferred in these patients for the treatment of severe chronic pain [10,12] and transmucosal products may be good candidates for the management of breakthrough pain [13,14].

The sublingual fentanyl tablet (Abstral^®^) is one of the transmucosal rapid onset opioids (ROOs). ROOs are well tolerated and may provide more efficacious treatment than oral morphine in patients suffering from breakthrough pain [14,15,16]. In clinical studies, patients experienced a significant pain relief after administration of sublingual fentanyl within 15 min [17]. Pharmacokinetic studies showed a fast increase in plasma concentrations after the administration of sublingual fentanyl with the first quantifiable drug concentrations (T_first_) found between 8 and 15 min, whereas the time to peak concentration (T_max_) varied from 30 min to 2 h [18,19]. Although a wide variation in pharmacokinetics is known for all fentanyl products, this variation is still largely unexplained [20]. Factors that might potentially influence absorption are of extra importance in transmucosal administration.

Currently, there are no data available on the use of sublingually delivered fentanyl in clinically relevant mucositis (grade 2 or higher) [6]. Therefore, it is unknown if mucositis influences the bioavailability of sublingually delivered fentanyl. A previous pilot study showed a trend towards a higher exposure to buccally delivered fentanyl in patients with mucositis compared to patients without mucositis [21], while another study showed no differences [22]. However, because of wide inter-individual variations in the pharmacokinetics of (transmucosal) fentanyl, cross-sectional studies may not be most appropriate to study effects of mucositis on fentanyl exposure. Therefore, we set up a prospective study in patients with head and neck cancer treated with chemo or bioradiotherapy.

As mentioned, xerostomia is another important side effect of chemo or bioradiotherapy and is mainly due to irradiation of the salivary glands. The severity of xerostomia is maximal at 6 weeks after the start of radiotherapy, but remains severe until 6 months after the last dose [23]. Because of the potential influence of xerostomia on the uptake of sublingually delivered fentanyl, we also investigated the systemic exposure to fentanyl six weeks after the end of the chemo or bioradiotherapy. At that time the intensity of xerostomia is still severe, but mucositis has resolved substantially in most patients [23,24].

## 2. Results

Fourteen patients were included of whom 13 patients (11 males and 2 females) were evaluable. One male patient was excluded for further analysis due to protocol violation by accidentally receiving fentanyl analgesic therapy outside the study protocol. The demographic data of these evaluable patients are presented in Table 1. The median age was 62 years (range 48 to 72). Patients were treated for cancer of the oropharynx (*n* = 4), hypopharynx (*n* = 4), larynx (*n* = 4), or combined oropharynx/larynx cancer (*n* = 1). Nine patients presented with cervical lymph node metastases. In all patients CTC grade ≥2 mucositis was diagnosed during the treatment with chemo or bioradiotherapy; in nine patients at 3 weeks, and in the other four patients at 6 weeks after the start of the treatment. The cumulative radiotherapy doses at T_1_ and T_2_ are given in Table 2.

### 2.1. Analyses for Mucositis

**Figure 1:**
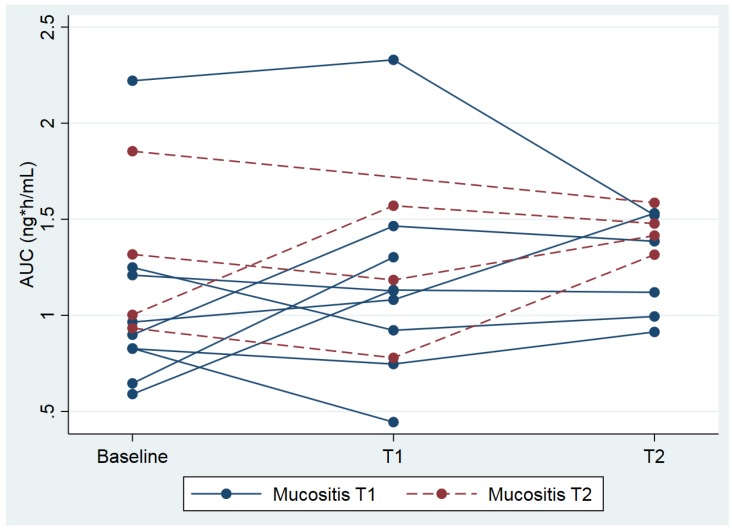
Individual area under the curve (AUCs) plotted against three time points; baseline, 3 weeks (T1) and 6 weeks (T2) after start. Each line represents an individual patient. Patients with a blue line had grade ≥2 mucositis at T1 and patients with a red line at T2.

**Figure 2:**
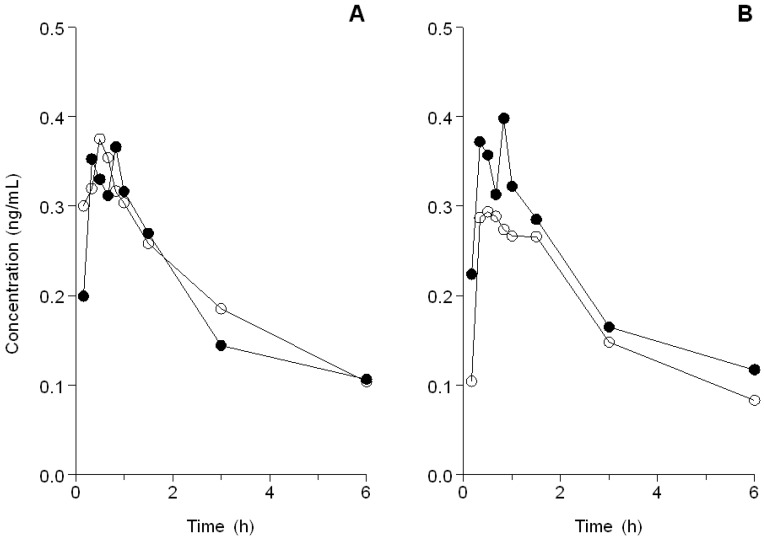
(**A**) The mean concentration time curve of fentanyl in patients with (o) and without (•) mucositis grade ≥ 2. (**B**) The mean concentration time curve of fentanyl in patients with (o) and without (•) xerostomia.

The geometric mean AUC_baseline_ was 1.04 ng/mL*h (coefficient of variation (CV) = 41.7%) and the AUC_mucositis_ was slightly higher: 1.18 ng/mL*h (CV = 36.1%). This was a relative difference of 12.7% (95% CI: −10.7% to +42.2%, *p =* 0.29; see Figure 1). The geometric mean of the maximum concentration (C_max_) of fentanyl at baseline was 0.43 ng/mL*h (CV = 40.0%), and C_max_ mucositis was 0.45 ng/mL*h (CV = 64.3%). This is a relative difference of 5.1% (95% CI: −28.1% to +53.8%, *p* = 0.78) (Figure 2A). The median time to reach C_max_ (T_max_) after administration of fentanyl was 40 min (range 10 min to 1 h and 35 min) at baseline, and T_max_ mucositis was 30 min (range 10 min to 1 h and 3 min), which did not differ significantly (*p* = 0.62).

### 2.2. Analyses for Xerostomia

Measurements at 6 weeks after finishing radiotherapy were available for eight patients. The other patients withdrew consent after finishing radiotherapy. In six out of eight evaluable patients, the GRIX score had increased for all four domains (Table 3). The geometric mean AUC of fentanyl at baseline in these eight patients was 1.12 ng/mL*h (CV = 45.1%) and at T_last_ this was 0.87 ng/mL*h (CV = 49.3%). This is a relative difference of −22.4% (95% CI: −51.9% to +25.3%, *p* = 0.25). The geometric mean of C_max_ of fentanyl at baseline was 0.44 ng/mL*h (CV = 44.4%) and at T_last_ this was 0.31 ng/mL*h (CV = 66.2%). This is a relative difference of −29.7% (95% CI: −64.6% to +39.7%, *p* = 0.27; see Figure 2B). T_max_ after administration of fentanyl was 40 min (range 20 min to 1 h and 35 min) at baseline, and 60 min (range 20 min to 1 h and 31 min) at T_last_, which did not differ significantly (*p* = 0.36).

### 2.3. Analysis of Pain

Pain was measured before every administration of the fentanyl. Only if pain scores were ≥4, pain scores were continued during sampling time. At 15 of the 52 pain measurements before administration of fentanyl pain intensity was ≥4. These 15 high pain scores occurred in 10 out of 13 patients. Three patients experienced pain at baseline, nine patients after onset of mucositis and three patients at T_last_. The median decrease in pain intensity after the administration of 200 μg fentanyl was 2 (range 0 to 8).

### 2.4. General Toxicity

Only two patients experienced dizziness and drowsiness CTC grade 1 after the administration of sublingual fentanyl. No other toxicities were seen due to administration of sublingual fentanyl.

## 3. Discussion

This is the first study that investigated the effects of mucositis and xerostomia on the pharmacokinetics of sublingually administered fentanyl in patients treated with chemo or bioradiotherapy for head and neck cancer. We found no significant differences in the exposure to sublingually delivered fentanyl in patients with a clinically relevant mucositis grade 2 or higher compared to their own baseline values. This is in line with two studies in patients with cancer that investigated the influence of mucositis (CTCAE grade 1) on buccally delivered fentanyl in an inter-patient comparison [21,22]. The major strengths of our study are the intra-patient comparisons and the standardized measurements in time.

Patients suffered from general erythema and oedema in the mouth, but not specifically under the tongue. Not all patients reached a dose of 39 Gy sublingually; the dose which has been correlated with severe mucositis (Table 3) [8]. The most severe mucositis is likely to occur in the radiotherapy area around the tumor (oropharynx, hypopharynx, and larynx) and the pathologic cervical lymph nodes. Therefore, the results of this study cannot be (simply) extrapolated to patients with moderate to severe mucositis caused by chemotherapy alone, since chemotherapy induced mucositis is typically located in the mouth and not only in the pharyngeal and laryngeal parts [25,26,27]. In addition, the within-patient variability in AUC was higher than expected beforehand, and therefore this outcome resulted in a lower power to detect a (potential) difference. This is a weakness of our study, and therefore the study may be assumed as a pilot study.

The increase in xerostomia we found after chemo or bioradiotherapy is in line with other studies [3,28,29]. Yet, our study was not powered to find significant differences in fentanyl pharmacokinetics between baseline and post-treatment measurements with xerostomia. We found 22% and 30% lower geometric means of respectively AUC and C_max_ during xerostomia compared to baseline. However, these differences were not statistically significant. Contrasting results were found in a study in patients with salivary gland hypofunction [30]. Moistening the mouth with water or pilocarpine hydrochloride (a cholinergic agonist), before taking sublingually delivered fentanyl, led to higher C_max_ and shorter T_max_ compared to the situation of xerostomia without moistening [30]. Our study results might be explained by moistening the mouth before every sublingual fentanyl administration.

Most patients were adequately treated with pain medication. Therefore, the number of measured episodes in which pain was assessed as ≥4 was low. The median decrease in pain intensity was 2, which is similar to studies on the use of sublingual fentanyl for breakthrough pain in patients with cancer [31,32]. This clinical effect is in line with the stable pharmacology we found during the chemo or bioradiotherapy. Although our patients suffered from mucositis, the results of sublingual fentanyl on pain seems to be comparable to all these studies.

In this study, sublingually delivered fentanyl was administrated to opioid naïve patients while it is registered for non-opioid naïve patients. Additionally, to ensure quantification of fentanyl plasma levels up to 8 h post dose, a higher dose of 200 mcg was given instead of the standard starting dose of 100 mcg. The higher starting dose did not led to any serious side effects in any of our opioid naïve patients.

Based on the findings in this study, we may provide some practical recommendations to physicians and patients. Sublingual fentanyl is a convenient option to treat breakthrough pain in patients with mucositis caused by chemo or bioradiotherapy. The uptake of sublingual fentanyl in patients with local ulcers or xerostomia is unknown and might be affected, and thus requires close monitoring of the effect. Moisturizing the mouth in case of xerostomia is recommended before the administration of fentanyl.

## 4. Methods

### 4.1. Patients

A single-center pharmacokinetic study was carried out at the Department of Medical Oncology of the Erasmus MC Cancer Institute between October 2014 and January 2017. The study was approved by the local medical ethics review board at 26th November 2013 and conducted in accordance with the latest version of the Declaration of Helsinki. The trial was registered at the Dutch Trial Registry (www.trialregister.nl ID: NTR4995). Patients of ≥18 years with head and neck cancer planned for curatively aimed radiotherapy with cisplatin or cetuximab were considered for inclusion in the study. Exclusion criteria included the use of fentanyl medication within one week before inclusion in the study (other opioids and non-opioid analgesics were allowed), opioid intolerance, former allergic reactions to opioids, serious psychiatric illness, confusion, intellectual disability, or earlier lymph nodes dissection in the head/neck region. The use of cytochrome P450 (CYP) inhibitors was allowed when there was no indication to change the dose of that drug during the study. Dexamethasone and aprepitant were allowed as standard anti-emetic therapy for patients treated with cisplatin. All enrolled patients provided written informed consent.

### 4.2. Study Design

Patients were given a single dose of 200 µg fentanyl (Abstral^®^) sublingually at 4 different time points in their regular treatment schedule of chemo or bioradiotherapy. Before administration of the fentanyl, patients had to rinse their mouth. The first dose of fentanyl was given before the start of the radiotherapy (baseline), the second dose 3 weeks after starting radiotherapy (T_1_), the third dose 6 weeks after starting radiotherapy (T_2_) and the last dose 6 weeks after finishing radiotherapy (T_last_). In case of chemo-radiotherapy, the fentanyl dose was planned 24 to 72 h before cisplatin treatment to avoid interference with the used CYP3A4 inhibiting or inducing antiemetic medication, e.g., aprepitant and dexamethasone. 

Radiotherapy consisted of 70 Gy in 35 fractions of 2 Gy to the primary tumor and clinically relevant positive nodes during a period of 6 to 7 weeks. Cisplatin (100 milligram per square meter (mg/m^2^) was given at day 1, 22, and 43 of the radiotherapy. Cetuximab (250 mg/m^2^) was given weekly during radiotherapy preceded by a loading dose (400 mg/m^2^) a week before start of the radiotherapy.

When patients needed analgesics, they could use all opioids except fentanyl products. When fentanyl was deemed necessary, patients left the study and were replaced.

### 4.3. Blood Sampling and Measurement of Fentanyl Concentrations

Pharmacokinetic (PK) samples were taken pre-dosing, and at 10, 20, 30, 40, 50, 60, 90, 180, and 360 min after administration of sublingual fentanyl.

Blood samples (4.5 mL) were collected in potassium ethylenediaminetetraacetic acid (EDTA) coated tubes and centrifuged for 10 min at 2500 to 3000× *g* at 4 °C. Plasma was transferred into polypropylene tubes (1.8 mL Nunc vials), which was stored at T < −70 °C (T < −20 °C during collection period) until the time of analysis. Fentanyl in plasma was quantitated using a validated UPLC-MS/MS method [33].

Pharmacokinetic data were analyzed by using Phoenix WinNonlin version 7.0 (Certara, Princeton, NJ, USA) to analyze concentration-versus-time data. Peak concentration (C_max_), time to peak concentration (T_max_), and area under the concentration-time curve (AUC) from 0 to 6 h after administration, were calculated.

### 4.4. Clinical Assessments

Mucositis, xerostomia, pain, and general toxicity were measured prior to the administration of sublingual fentanyl. Mucositis was scored with CTCAE 4.03 toxicity criteria [6], xerostomia with the Groningen Radiotherapy-induced Xerostomia questionnaire (GRIX) [28] and pain with the Numerical Rating Scale (NRS) [34]. When patients suffered from moderate-severe pain (NRS ≥ 4) at the start of the PK sampling, then pain was also assessed at the PK sampling time points. 

Other toxicities, i.e., nausea, vomiting, anorexia, dizziness, drowsiness, and fatigue, were also scored with CTCAE 4.03 prior to, and one hour after, the administration of sublingual fentanyl.

### 4.5. Statistical Considerations

#### 4.5.1. Sample Size Calculation

The primary outcome measure was fentanyl AUC. A relative difference of 25% between the AUC at day 1 (AUC_baseline_) and the AUC at the first moment a mucositis with a severity of at least CTC grade 2 (AUC_muco_) was found during chemo of bioradiotherapy was considered as clinically relevant. Assuming a within-patient variability in AUC of 20%, 13 evaluable patients were needed to obtain 80% power (2-sided significance level α = 0.05) to detect a difference [35].

#### 4.5.2. Statistical Analyses

The difference in AUC between day 1 and the first moment with a mucositis grade ≥2 (for each individual patient determined) was analyzed by means of a paired *t*-test. Since it was assumed that the AUC follows a log-normal distribution, analyses were performed on log-transformed data. The results were then back-transformed by taking the exponentials from the difference and corresponding 95% confidence interval, which represents the ratio of the geometric means and can be interpreted as the percentage of change (i.e., relative difference (RD)) between AUC_muco_ and AUC_baseline_. A similar approach was used for the analysis of C_max_. Differences in T_max_ were analyzed using a Wilcoxon signed rank test.

For the analysis of the effect of xerostomia on the PK of sublingual fentanyl, differences in AUC, C_max_, and T_max_ were analyzed in the same way as the analysis for mucositis.

## 5. Conclusions

Mucositis grade 2 or higher, caused by radiotherapy in combination with cisplatin or cetuximab, did not significantly influence the systemic exposure to sublingually delivered fentanyl. Xerostomia led to non-significant lower AUC values (30%) and fentanyl concentrations (22%) compared to baseline. Therefore, patients with pain during and after chemo or bioradiotherapy for head and neck cancer may be safely treated with sublingually delivered fentanyl.

## Figures and Tables

**Table 1:** Patient characteristics.

Variable	Total *N* = 13
Sex, n (%)	
Male	11
Female	2
Age, years (median and range)	62 (48–72)
BMI (median and IQR)	25.4 (22.8–26.9)
Tumor type	
oropharyngeal carcinoma	4
hypopharyngeal carcinoma	4
laryngeal carcinoma	4
combined oropharyngeal and laryngeal carcinoma	1
Concurrent to radiotherapy	
Cisplatin	5
Cetuximab	8
Laboratory results (median (IQR) (normal range)	
Creatinine (55–90 µL/min)	79.0 (78.0–90.0)
MDRD (>60 mL/min/1.73 m^2^)	82.5 (73.0–87.5)
AST (<31 U/L)	24.5 (22.0–34.0)
ALT (<34 U/L)	40.5 (21.0–48.0)
Bilirubin (<17 µmol/L)	6.0 (5.0–7.0)
Albumin (35–50 g/L)	41.5 (41.0–46.0)
ALP (<98 U/L)	80.5 (62.0–100.0)

Legend: Abbreviations: BMI; body mass index, IQR; interquartile range, MDRD; modification of diet in renal disease, AST; aspartate aminotransferase, ALT; alanine aminotransferase, ALP; alkaline phosphatase.

**Table 2:** Radiotherapy dose during the chemo- or bioradiotherapy.

Radiotherapy Dose and Fentanyl AUC	T_baseline_	T_1_	T_2_	T_mucositis_ T1 *n* = 9	T_mucositis_ T2 *n* = 4
Radiotherapy dose sublingual in Gy (mean, SD)	-	13.2 (6.7)	28.2 (12.9)	12.9 (4.4)	40.1 (SD 13.7)
Radiotherapy dose total in Gy (mean SD)	-	30.8 (6.2)	55.6 (4.7)	32.7 (6.7)	54.3 (1.3)
Fentanyl AUC ng/mL*h geometric mean (CV %)	1.04 (41.7)	1.09 (40.6)	1.31 (42.2)	x	x

Legend: T_1_ = 3 w after start of radiotherapy; T_2_ = 6 w after start of radiotherapy.

**Table 3:** Xerostomia analysis measured by GRIX.

GRIX Score	Baseline (*n* = 8) 0–100	T_last_ (*n* = 8) 0–100
Day xerostomia Median (IQR)	11.11 (0.00–22.22)	38.89 (22.22–77.78)
Day sticky saliva Median (IQR)	0.00 (0.00–11.11)	27.78 (0.00–61.11)
Night xerostomia Median (IQR)	22.22 (5.56–27.78)	38.89 (33.33–66.67)
Night sticky saliva Median (IQR)	0.00 (0.00–8.33)	16.67 (0.00–66.67)

Legend: IQR: interquartile range.
